# High-resolution motion compensated MRA in patients with congenital heart disease using extracellular contrast agent at 3 Tesla

**DOI:** 10.1186/1532-429X-14-75

**Published:** 2012-10-29

**Authors:** Darius Dabir, Claas Philip Naehle, Ralf Clauberg, Juergen Gieseke, Hans H Schild, Daniel Thomas

**Affiliations:** 1 Department of Radiology, University of Bonn, Bonn, Germany; 2 Philips Healthcare, Hamburg, Germany

**Keywords:** Congenital heart disease, Magnetic resonance angiography, 3 Tesla, High-resolution, Thoracic vasculature, Extracellular contrast agent, ECG-gated, Navigator-gated, Gadobutrol

## Abstract

**Background:**

Using first-pass MRA (FP-MRA) spatial resolution is limited by breath-hold duration. In addition, image quality may be hampered by respiratory and cardiac motion artefacts. In order to overcome these limitations an ECG- and navigator-gated high-resolution-MRA sequence (HR-MRA) with slow infusion of extracellular contrast agent was implemented at 3 Tesla for the assessment of congenital heart disease and compared to standard first-pass-MRA (FP-MRA).

**Methods:**

34 patients (median age: 13 years) with congenital heart disease (CHD) were prospectively examined on a 3 Tesla system. The CMR-protocol comprised functional imaging, FP- and HR-MRA, and viability imaging. After the acquisition of the FP-MRA sequence using a single dose of extracellular contrast agent the motion compensated HR-MRA sequence with isotropic resolution was acquired while injecting the second single dose, utilizing the timeframe before viability imaging. Qualitative scores for image quality (two independent reviewers) as well as quantitative measurements of vessel sharpness and relative contrast were compared using the Wilcoxon signed-rank test. Quantitative measurements of vessel diameters were compared using the Bland-Altman test.

**Results:**

The mean image quality score revealed significantly better image quality of the HR-MRA sequence compared to the FP-MRA sequence in all vessels of interest (ascending aorta (AA), left pulmonary artery (LPA), left superior pulmonary vein (LSPV), coronary sinus (CS), and coronary ostia (CO); all p < 0.0001). In comparison to FP-MRA, HR-MRA revealed significantly better vessel sharpness for all considered vessels (AA, LSPV and LPA; all p < 0.0001). The relative contrast of the HR-MRA sequence was less compared to the FP-MRA sequence (AA: p <0.028, main pulmonary artery: p <0.004, LSPV: p <0.005). Both, the results of the intra- and interobserver measurements of the vessel diameters revealed closer correlation and closer 95 % limits of agreement for the HR-MRA. HR-MRA revealed one additional clinical finding, missed by FP-MRA.

**Conclusions:**

An ECG- and navigator-gated HR-MRA-protocol with infusion of extracellular contrast agent at 3 Tesla is feasible. HR-MRA delivers significantly better image quality and vessel sharpness compared to FP-MRA. It may be integrated into a standard CMR-protocol for patients with CHD without the need for additional contrast agent injection and without any additional examination time.

## Background

Cardiovasculars magnetic resonance (CMR) has become the main imaging modality for the assessment of congenital heart disease (CHD) in patients in whom echocardiography is limited because of a restricted acoustic window. Furthermore, magnetic resonance angiography has become a mainstay of CHD imaging, because it allows for accurate non-invasive assessment of the arterial and venous thoracic vasculature. At present, standard first-pass(FP)-MRA of the thoracic vasculature is performed using an extracellular contrast agent and a single or multiphase FP-MRA sequence. FP-MRA is performed without cardiac gating and during one breath-hold [1,2]. Consequently, spatial resolution is limited by breath-hold duration and cardiac motion artefacts may lead to vessel blurring.

Recent studies at 1.5 Tesla (T) have shown that the extended plasma half-life of a blood-pool contrast agent (BP-CA) allows for high-resolution respiratory navigator- and ECG-gated MRA (HR-MRA) during the steady state thereby overcoming the aforementioned limitations. In comparison to FP-MRA, both image quality and vessel sharpness were significantly improved using the gated HR-MRA approach [3]. However, blood-pool contrast agents do not allow for viability imaging. At the same time, the value of viability imaging in CHD patients has been demonstrated and it has now become an integral part of CMR imaging of CHD at most institutions today [4,5].

Contrast enhanced CMR benefits from a transition to a higher field strength due to an almost unchanged T1-relaxivity for extracellular contrast agents and an increased T1-relaxivity for stationary tissue, resulting in a higher contrast to noise ratio (CNR) [6]. Taking advantage of the higher CNR, a recent study demonstrated the feasibility of coronary MRA during slow infusion of an extracellular contrast agent at 3 Tesla [7].

We hypothesize that a high-resolution respiratory navigator- and ECG-gated MRA technique using an extracellular contrast agent during slow infusion at 3 Tesla may be used for imaging of the thoracic vasculature while still allowing for viability imaging.

Thus, the aim of the study was to implement an HR-MRA-protocol covering the entire thoracic vasculature using a standard extracellular contrast agent (Gadobutrol) and to compare it to a standard FP-MRA sequence in patients with CHD.

## Methods

### Study population

Patients with CHD were consecutively enrolled in this prospective study. Patients with contraindications to CMR [8], however, were excluded. The study protocol was approved by the local institutional review board. All patients or their respective legal guardians gave their written informed consent.

### CMR protocol

The studies were performed using a clinical whole-body 3.0 T scanner (Achieva Tx, Philips Healthcare, Best, the Netherlands) with a maximum gradient amplitude of 80 mT/m and a maximum slew rate of 200 (mT·m^-1^)/ms. For signal reception, a 6-channel cardiac phased-array surface coil was used. All patients were examined in the supine position. Before the examination, a 20-gauge intravenous catheter was placed in an antecubital vein to allow for contrast medium injection. Depending on the age of the individual patient, the examinations were performed under general anaesthesia.

The MRA sequences were part of a standard protocol for the evaluation of CHD. The FP-MRA was performed after acquisition of functional cine images in the standard cardiac axes, as well as flow measurements if necessary. Immediately after acquisition of the FP-MRA, the HR-MRA was obtained during the waiting period for the delayed enhancement sequence. Therefore, the overall examination time was not increased.

For the multiphase FP-MRA, a standard T1-weighted 3D gradient echo sequence with a centric ordered k-space acquisition (CENTRA) scheme in both slice- and phase-encoding direction was used. The sequence was acquired during breath-hold and started after injection of gadobutrol at a dose of 0,1 mmol/kg bodyweight and at a rate of 1,5 ml/s, followed by a 20 ml saline flush using the same injection rate. For the injection a commercial power injector (Spectris, Medrad, Volkach, Germany) was used. Detailed sequence parameters were as follows: TR/TE = 5.0/1.66; flip angle = 30°; 4 mm coronal overcontiguous partitions were reconstructed to 60 contiguous 2 mm slices; acquisition matrix = 240 x 240; field of view (FOV) = 360 mm, resulting in an acquired in-plane resolution of 1.5 x 1.5 mm.

The isotropic HR-MRA was initiated during injection of gadobutrol at a dose of 0.1 mmol/kg bodyweight and at a rate of 0.3 ml/s, followed by a 20 ml saline flush using the same injection rate. The HR-MRA consisted of a single-phase T1-weighted 3D inversion recovery sequence. Initial tests before initiation of the study revealed that an inversion time (TI) of 280 ms revealed the most sufficient background suppression while still maintaining an overall high signal. The pulse sequence was ECG-gated to end-diastole to minimize cardiac motion. The k-space acquisition scheme was “high-low”. Depending on the patients′ heart rate, the acquisition time was set between 100–120 ms. Respiratory navigator gating was used for data acquisition during free breathing. Additional imaging parameters were as follows: TR/TE 3.2/1.05 ms, flip angle = 20°, 2.4 mm coronal overcontiguous partitions were reconstructed to 80 contiguous 1.2 mm slices; acquisition matrix 300 x 298, FOV = 360 mm, yielding an acquired in-plane resolution of 1.2 x 1.2 mm.

For both, FP- and HR-MRA, one signal average and parallel imaging (SENSE) with a factor of four were used.

### Image analysis

#### Qualitative analysis

MRA-images were evaluated separately by two experienced readers (View Forum 5.22, Philips, Best, the Netherlands) regarding image quality and additional clinical findings. Both readers were aware of the main diagnosis and major surgical procedures in each patient, but were blinded to the results of the second reader. FP-MRA and HR-MRA were evaluated on separate days (≥ 7 days between measurements).

### Image quality

Vessels of interest were the aortic arch (AA), left superior pulmonary vein (LSPV), left pulmonary artery (LPA), coronary sinus (CS), and coronary ostia (CO). Image quality was rated using maximum intensity projections (MIPs) of both types of MRA based on a five-point grading scale with respect to image artefacts (e.g. breathing artefacts and cardiac motion artefacts) and anatomic delineation (i.e. border definition). The rating scale was as follows: 1) excellent image quality (no artefacts, good delineation of the vessel border); 2) above average image quality (very few artefacts, very little blurring of the vessel border); 3) average image quality (some artefacts, some vessel blurring); 4) poor image quality (severe artefacts, severe blurring of the vessel border); 5) non diagnostic image quality.

### Quantitative analysis

Vessel sharpness, relative contrast, and intraobserver measurements (≥ 7 days between measurements) were performed by the first reviewer. Measurements of vessel diameters for the assessment of interobserver variability were performed by the first and second reviewer.

### Vessel sharpness

In order to determine vessel sharpness, an intensity profile along a specified segment in both types of MRA of the representative vessels was created with a public domain image processing software (ImageJ, U.S. National Institutes of Health, Bethesda, Maryland). The distance between 20% and 80% of maximum intensity was measured for each side of the vessel and then averaged. The reciprocal of the averaged distance was taken as sharpness, where a greater value is consistent with a better vessel definition.

### Relative contrast

Parallel imaging, employed for both MRA sequences did not allow for absolute quantitative measurements of signal- and contrast-to-noise. Therefore, contrast ratios (RC) between vessels (SV) and signal in the surrounding tissue (ST) were calculated based on the following formula: $RC=\frac{\left(SV-ST\right)}{\left(SV+ST\right)}$

Regions of interest (> 100 mm^2^) were placed in the respective vessels (AA, main pulmonary artery (PA), LSPV) and the adjacent tissue. The dynamic phase exhibiting the strongest enhancement in the vessel of interest was chosen for the FP-MRA measurements. For both, the FP- and the HR-MRA, identical locations for the measurements were chosen.

### Inter- and intraobserver agreement

Cranio-caudal and anterior-posterior diameters of AA, LPA, and LSPV were measured in both, FP- and HR-MRA sequences.

### Statistical analysis

The data analysis was performed using commercially available software (Analyze-it for Microsoft Excel v. 2.12; Analyze-it Software, London, UK). All data are given as mean ± standard deviation (SD). Qualitative scores for image quality as well as quantitative measurements of vessel sharpness and relative contrast were compared using the Wilcoxon signed-rank test. Quantitative measurements of vessel diameters were compared using the Bland-Altman test in order to evaluate agreement.

## Results

A total of 34 patients (18 male, 16 female) with a mean age of 18,1 years (median 13 years) were enrolled in the study (Table 1). The study protocol was successfully performed in all patients. The duration of the FP-MRA sequence was 15 seconds per dynamic scan (a total of three dynamics was acquired). The mean duration of the HR-MRA sequence was 2 minutes and 18 seconds ± 29 seconds (range from 1:38 to 3:40 minutes).

**Table 1:** Indication for CMR

**Indication for CMR**	**Number of patients**
Follow up of tetralogy of Fallot	6
Follow up of aortic isthmus stenosis	5
Follow up of transposition of the great arteries	3
Follow up of double outlet right ventricle	3
Follow up of common arterial trunk	2
Loeys-Dietz/Marfan-syndrome	3
Follow up of aortal valve stenosis	2
Pulmonary stenosis	2
ASD/VSD	2
Follow up of pulmonary atresia	1
Left atrial appendage aneurysm	1
Follow up of patent ductus arteriosus	1
Ebstein anomaly	1
Other	2

### Image quality

The mean image quality score, independently rated by both reviewers, revealed significantly better image quality of the HR-MRA sequence compared to the FP-MRA sequence in all vessels of interest (p < 0.0001):

AA: FP-MRA 2.78 ± 0.48 (median: 3; range: 2–4) vs. HR-MRA 2.26 ± 0.62 (median: 2; range: 1–4), LSPV: FP-MRA 3.19 ± 0.50 (median: 3; range: 2–4) vs. HR-MRA 2.38 ± 0.62 (median: 2; range: 1–4), LPA: FP-MRA 3.06 ± 0.46 (median: 3; range: 2–4) vs. HR-MRA 2.47 ± 0.61 (median: 2; range: 2–4); CS: FP-MRA 3.84 ± 0.68 (median: 4; range: 3–5) vs. HR-MRA 2.50 ± 0.74 (median: 2; range: 2–5) and CO: FP-MRA 4.47 ± 0.63 (median: 5; range: 3–5) vs. HR-MRA 3.10 ± 1.01 (median: 3; range: 2–5).

**Figure 1:**
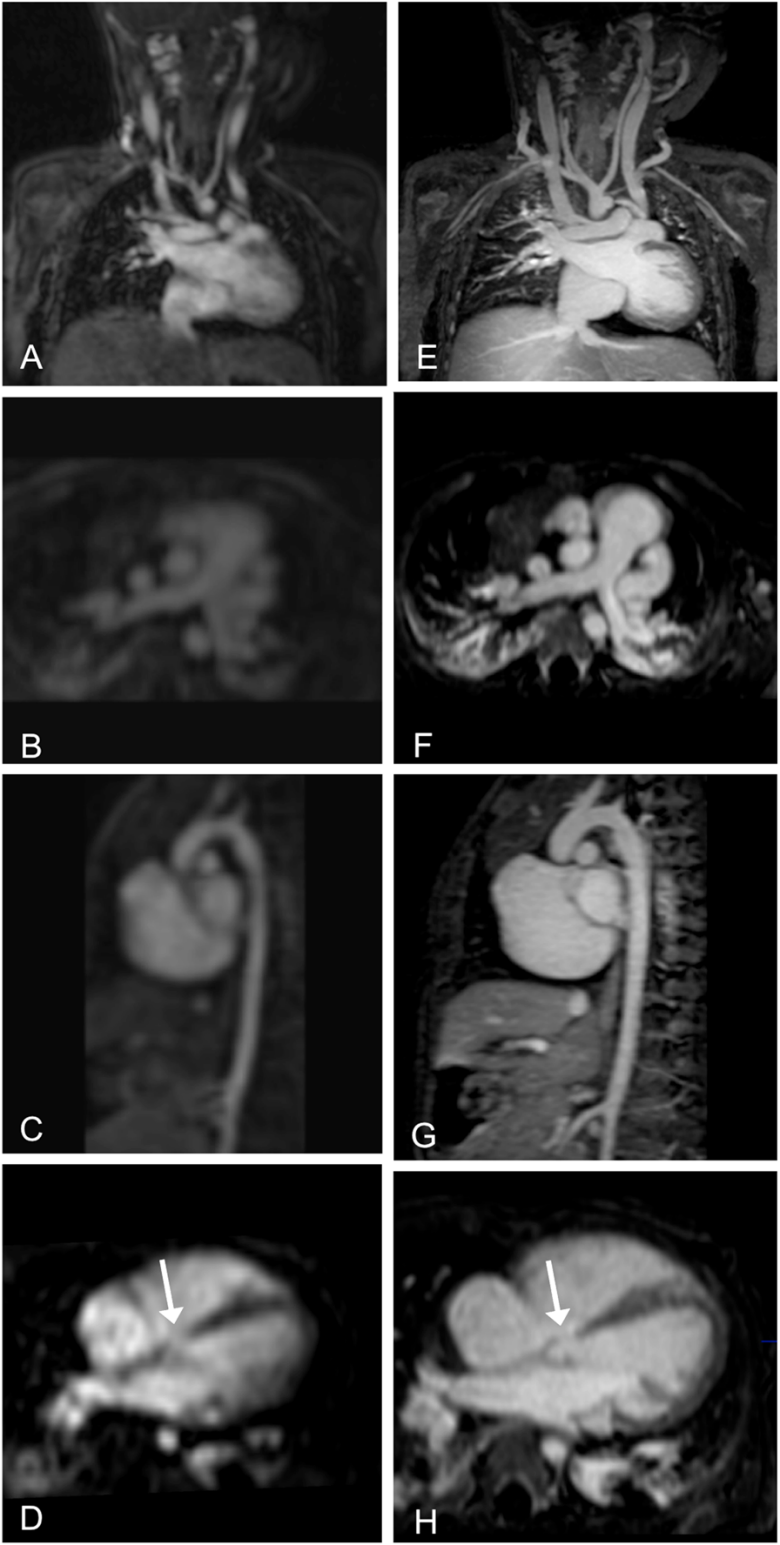
**10-month-old patient with ventricular septal defect (arrow), persistent left superior vena cava, bilateral atelectasis and bovine arch.** FP-MRA (**A**-**D**) and HR-MRA (**E**-**H**) in coronal (**A**,**E**), axial (**B**,**F**,**D**,**H**) and sagittal plane (**C**,**G**).

**Figure 2:**
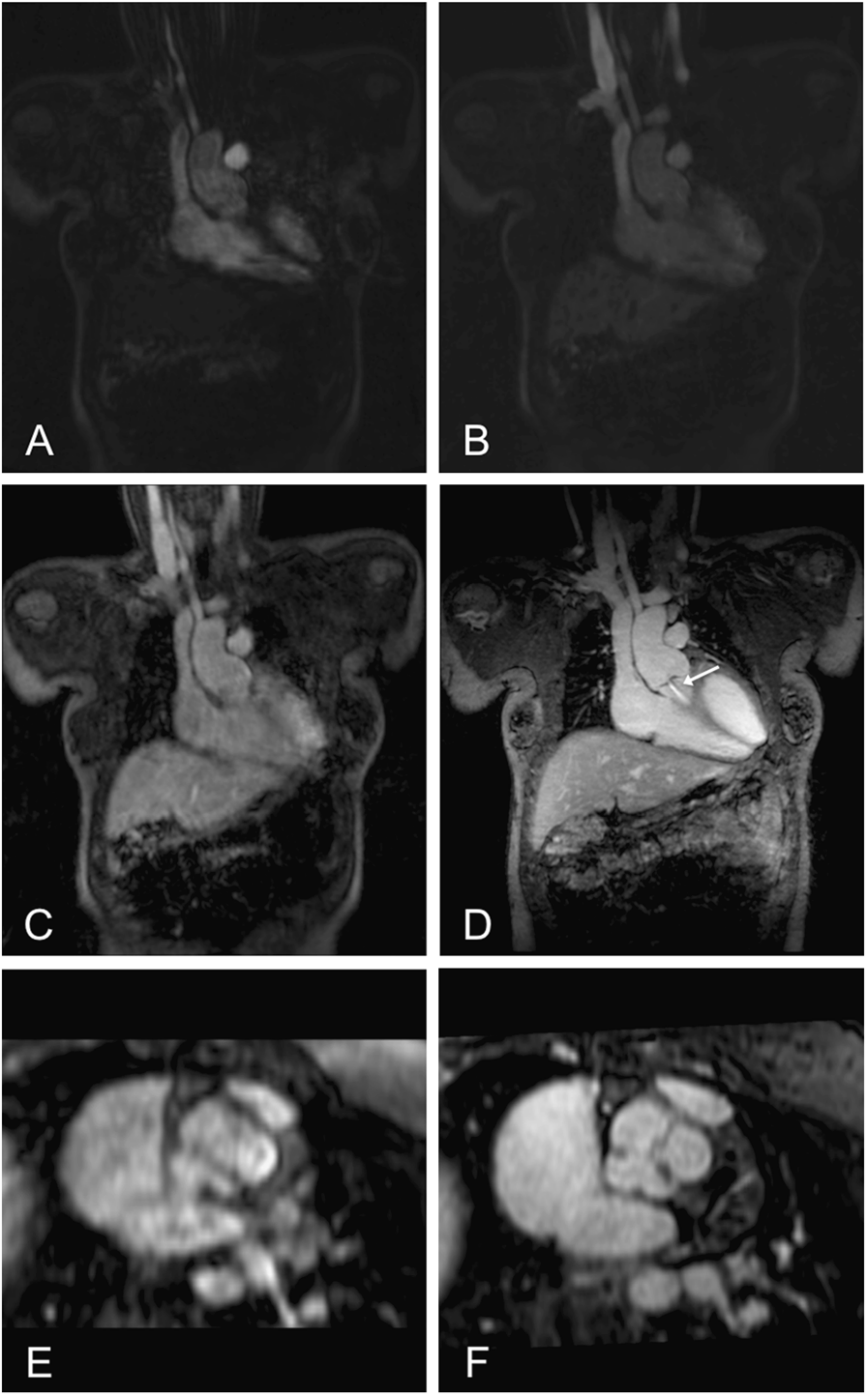
**11-year-old patient after surgical correction of a common arterial trunk.** FP-MRA (**A-C**) and HR-MRA (**D**) in coronal plane. Note the insufficiency jet of the aortic valve (arrow). En face view of the arterial truncal valve with three leaflets in FP- (**E**) and HR-MRA (**F**).

**Figure 3:**
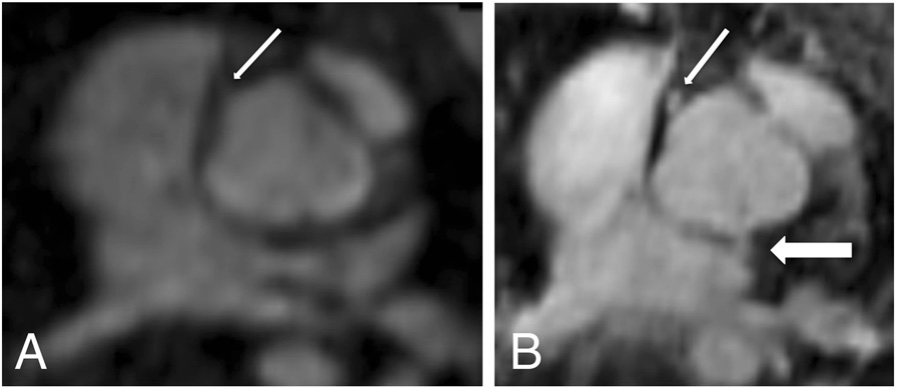
**11-year-old patient after surgical correction of a common arterial trunk (same patient as in Figure**2**).** Axial plane through the ascending aorta at the level of the coronary ostia in FP-MRA (**A**) and HR-MRA (**B**). Whereas in the FP-MRA only the origin of the right coronary artery can be detected (thin arrow), the HR-MRA accurately depicts the origins of both coronary arteries (thin arrow: right coronary artery; thick arrow: left coronary artery) revealing an abnormal origin of the left coronary artery out of the non-coronary cusp.

Typical examples demonstrating the superior image quality of HR-MRA compared to FP-MRA are given in Figures 1, 2, and 3. HR-MRA revealed one clinical finding that was missed by FP-MRA: Due to cardiac motion, an abnormal offspring of the left coronary artery could only be detected by HR-MRA (Figure 3).

### Vessel sharpness

In comparison to the FP-MRA sequence, the HR-MRA sequence revealed a significantly better vessel sharpness for all considered vessels:

AA: FP-MRA: 0.49 ± 0.09 vs. HR-MRA: 0.70 ± 0.13, LSPV: FP-MRA: 0.38 ± 0.10 vs. HR-MRA: 0.58 ± 0.12, LPA: FP-MRA: 0.36 ± 0.06 vs. HR-MRA: 0.51 ± 0.12 (all p < 0.0001).

### Relative contrast

The relative contrast of the HR-MRA sequence was less compared to the FP-MRA sequence:

AA: FP-MRA: 0.84 ± 0.06 vs. HR-MRA: 0.81 ± 0.07 (p <0.028), PA: FP-MRA: 0.87 ± 0.05 vs. HR-MRA: 0.84 ±0.06 (p <0.004), LSPV: FP-MRA: 0.86 ± 0.06 vs. HR-MRA: 0.83 ± 0.06 (p <0.005).

### Intra- and interobserver agreement

**Figure 4:**
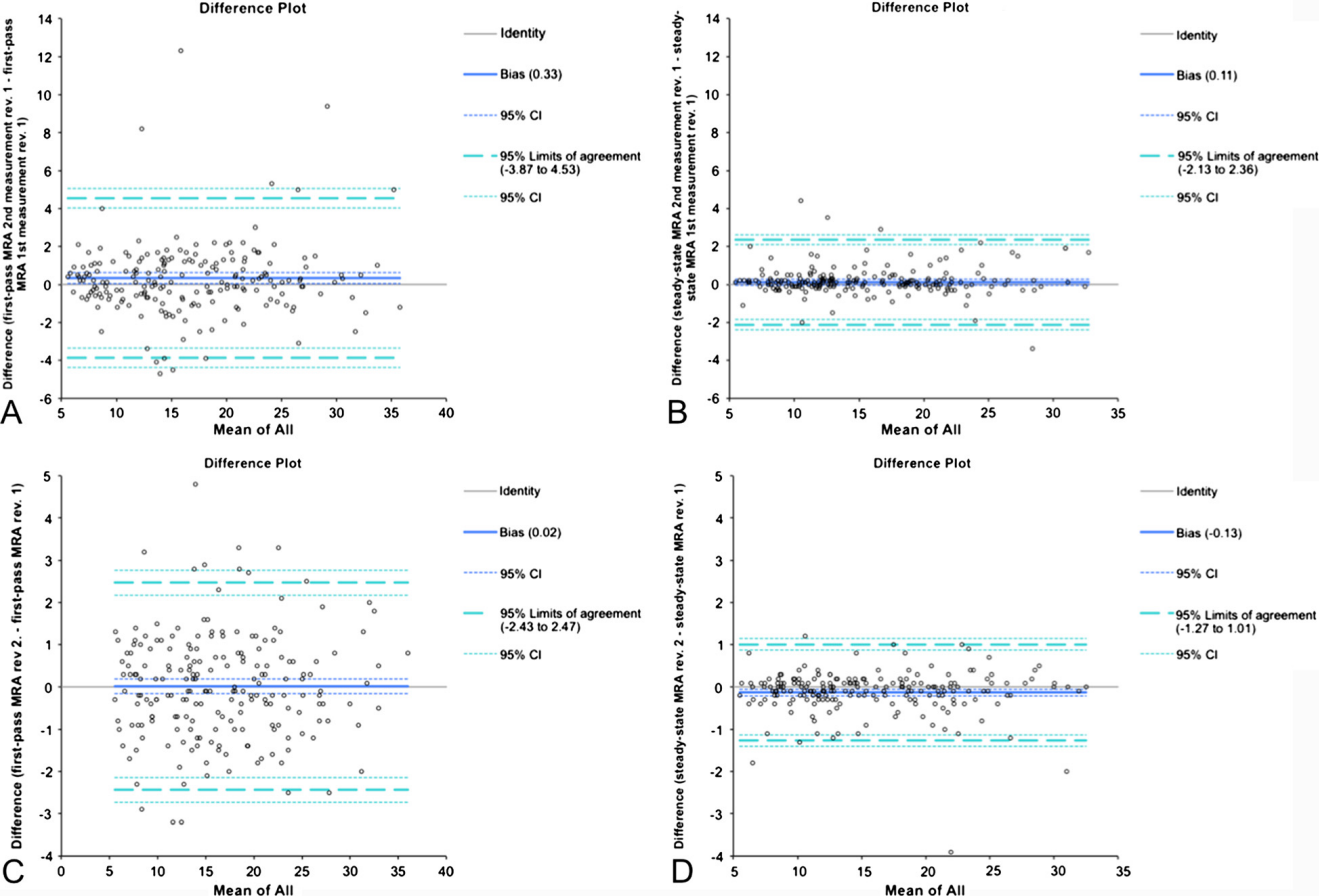
**Intraobserver method agreement of FP-MRA- (A) and HR-MRA-measurements (B) of reviewer 1.** Interobserver method agreement derived from FP-MRA (**C**) and HR-MRA (**D**) measurements of reviewers 1 and 2.

Both, the results of the intra- and interobserver measurements of the vessel diameters revealed much closer 95% limits of agreement and higher correlation coefficients for the HR-MRA sequence in comparison to the FP-MRA sequence (Figure 4).

## Discussion

Cardiac CMR, especially as a postoperative follow up modality, has become the primary diagnostic imaging tool for CHD patients. Not only because it is the gold standard for the evaluation of left and right ventricular function, but also because it allows for reliable assessment of vascular structures independent of an acoustic window. While recent studies have mostly focused on increasing the temporal resolution of MRA in patients with CHD [9,10], imaging issues related to motion artefacts (respiratory and cardiac motion) have yet not been fully addressed. Especially in children and diseased patients limited compliance may deteriorate image quality of FP-MRA.

The combination of ECG- and respiratory navigator-gated MRA using a blood-pool contrast agent at 1.5 T has shown to overcome these limitations [3]. However, blood-pool contrast agents are of higher costs and even more importantly, they exclude the possibility of viability imaging. Studies have revealed the value of viability imaging in patients after surgery of CHD as the presence of scar or fibrous tissue could be related to adverse ventricular mechanics and a higher prevalence of non-sustained ventricular tachycardia [4,5].

Thus, an ideal imaging protocol for the assessment of complex congenital heart disease should not only include functional imaging and MRA but also viability imaging. In this study, a comprehensive imaging protocol was established, which includes functional imaging, FP-MRA, as well as viability imaging. Adding viability imaging does require the injection of a double dose of contrast agent as well as a waiting period of approximately 12–15 minutes after contrast injection to allow for delayed enhancement imaging. The second injection and waiting period were used to acquire a high-resolution motion compensated MRA sequence (HR-MRA) at no cost of additional scan time or contrast agent.

In comparison to the FP-MRA, the HR-MRA sequence revealed a significantly better image quality and vessel sharpness (Figures 1, 2, 3). Most likely, these results could be attributed to the higher resolution as well as the improved motion compensation through ECG-gating and navigator respiratory compensation. Another noteworthy advantage of HR-MRA compared to FP-MRA is the acquisition of isotropic voxels allowing for multiplanar image reconstruction without any loss of image resolution (Figure 1, 2). The fact that HR-MRA revealed an abnormal offspring of the left coronary artery, missed by FP-MRA in one patient, highlights the value of isotropic high resolution and motion compensation (Figure 3). One inherent drawback of the motion compensated approach is the missing dynamic information. Thus, it can only be used as an add on sequence. However, implementing the HR-MRA into a standard CHD-CMR-protocol does not involve any additional scan time or the use of additional contrast agent injection.

Due to the slow injection rate that was used for the HR-MRA, the relative contrast between the examined vessels and surrounding tissue in all acquired HR-sequences was lower compared to the bolus technique (FP-MRA). Due to the most compact bolus geometry in the PA during the first-pass, the largest difference between relative contrasts was found in this vessel.

Intra- and interobserver agreement results revealed much closer 95% limits of agreement and higher correlation coefficients for the HR-MRA sequence in comparison to the FP-MRA sequence (Figure 4). This shows the importance of vessel sharpness rather than relative contrast for accurate assessment of vessel diameters.

The MRA sequence employed in this study was similar to the approach by Naehle et al. [3]. They compared a first-pass MRA-protocol with a high-resolution motion compensated steady-state protocol following the injection of a blood-pool contrast agent (Vasovist) at 1.5 T [11]. The use of a blood-pool agent in their study did allow them to deliberately choose an inversion time for optimal suppression of background tissues, whereas in our study a fixed inversion time had to be used due to the limited acquisition window after contrast agent injection. Also, the spatial resolution had to be sacrificed to allow for faster acquisition of the 3D HR-MRA dataset. Nevertheless, vessel sharpness of the motion compensated MRA vs. the first-pass-MRA was very similar in both studies, using the same measurement technique. Similar to Naehle et al., an additional clinical finding was also detected by motion compensated MRA which was missed by FP-MRA, underscoring the value of motion compensation. Most importantly, the HR-MRA approach in this study overcomes a major limitation of the previously published approach as it allows for viability imaging and thus fits perfectly into a standard CHD-CMR-protocol. In addition, contrary to blood-pool contrast agents, the contrast agent used in this study has recently been approved for use in children > 2 years in North America and Europe.

Previous studies have demonstrated the value of 3D whole-heart imaging in pediatric populations using balanced steady-state free precession (b-SSFP) sequences [12,13]. However, one advantage of the proposed approach in comparison to b-SSFP is the high CNR, which is achieved through the use of an IR-pulse suppressing the background signal. In addition, while the non-contrast enhanced b-SSFP approach has proven to work at 1.5 T, its use may be limited by artefacts related to b0 and b1 field inhomogeneity at 3 Tesla.

Another recent study by Yang et al. [7] demonstrated the feasibility and diagnostic yield of a contrast-enhanced whole-heart coronary MRA at 3 T using an extracellular contrast agent (gadobenate dimeglumine) during slow injection. Interestingly, although the contrast agent in this study (gadobutrol) has an even shorter plasma half-life than gadobenate dimeglumine, the acquisition of a 3D motion compensated HR-MRA dataset, covering the entire thorax with an even higher resolution, was feasible.

Using a fixed TI of 200 ms, Yang et al. achieved an optimal suppression of background tissue. In contrast, the TI chosen in this study was significantly longer for several reasons. First, the contrast agent could not be completely injected as a slow infusion, because a bolus injection is required for FP-MRA, which in turn results in a broader variation of contrast agent concentration in the blood. Second, the albumin binding of gadobutrol is even less, yielding an even shorter imaging window. Finally, the heart rate of the patients in this study was not controlled resulting in a wide range of heart rates. Thus, we decided to choose a longer TI, which might not reveal optimal suppression of background tissue but yields a good overall signal to noise ratio for the majority of the patients. Moreover for imaging of the entire thoracic vasculature, suppression of background signal is less critical compared to coronary imaging.

Due to variations in patients’ total amount of contrast agent injected, variation in heart rate, navigator performance and the subsequent variation of acquisition duration, it is difficult to determine the optimum contrast agent injection protocol for the proposed FP/HR-MRA approach. The use of a bolus for FP-MRA and subsequent slow injection of contrast agent has shown to deliver a good CNR in this study. However, further research is warranted to determine, whether a further improvement of CNR can be achieved with a tailored protocol taking into account the aforementioned variables.

## Conclusions

In conclusion, it could be shown that an ECG- and navigator-gated HR-MRA-protocol with infusion of extracellular contrast agent at 3 Tesla is feasible. Furthermore, it delivers significantly better image quality and vessel sharpness at nearly equivalent contrast compared to FP-MRA. Therefore, it may be integrated into a standard CMR-protocol for patients with CHD without the need for additional contrast agent injection and without any additional examination time.

## Competing interests

The authors declare that they have no competing interests. JG is an employee of Philips Healthcare.

## Authors’ contribution

DD was responsible for design of the study, patient recruitment, MRI study reading, data analysis and manuscript drafting. CN participated in study design and statistical analysis. RC contributed to patient examination and data analysis. JG participated in sequence optimisation. HS made substantial contributions to the conception and design of the study. DT was responsible for design of the study, patient recruitment, MRI study reading, data analysis and manuscript drafting. All authors read and approved the final manuscript.
